# Supplementary material for: A systematic review of psychosocial functioning changes after gender-affirming hormone therapy among transgender people
Source: Nat Hum Behav. 2023 May 22;7(8):1320–31. doi: 10.1038/s41562-023-01605-w (PMC10444622; doi:10.1038/s41562-023-01605-w)
Supplement: Supplementary file 4 — Supplementary table with study coding. [file 41562_2023_1605_MOESM4_ESM.pdf]

Supplementary Table 5. Studies included in systematic review by study type (K = 46)

Author	Aim	Design	Measures	Number of participants	Participant demographics	Results	Location
Blanchard et al (1983)	Examined the relationship between psychological and social adjustment, and gender transition.	Cross-sectional	Tension (TSC-VII)<br>Depression (TSC-IV)<br>Involvement<br>Cohabitation	55	Transgender women	Social feminization (accessing GAHT) led to significantly reduced feelings of tension and depression.	Canada
Bouman et al (2016)	Investigated the sociodemographic and clinical characteristics of older (50> YOA) trans people attending a national service and to investigate the influence of cross-sex hormones (CHT) on psychopathology.	Cross-sectional	Hospital anxiety and depression scale (HADs)<br>Rosenberg Self-esteem scale<br>Experiences of transphobia scale<br>Multidimensional Scale of Perceived Social Support<br>Inventory of interpersonal problems<br>Non-suicidal self-injury questionnaire	74	Transgender women (71)<br>Transgender men (3)	Transgender people on hormone therapy reported significantly lower levels of socialization problems, anxiety and depression as well as higher levels of self-esteem.	UK
Butler et al (2019)	Examined whether gender-affirming medical interventions were associated with lower social anxiety among transgender individuals.	Cross-sectional	Mini-Social Phobia Inventory (social anxiety) (Mini-SPIN)<br>Gender affirming medical interventions (GAMI)	715	Transgender people who completed the Trans Health Survey<br>(291 Transfeminine, 424 Transmasculine)	Individuals who had completed a course of hormones reported significantly lower MINI-SPIN (social anxiety) scores compared to those who were planning on using hormones.	USA

Davis & Keo-Meier (2014)	Examined the effects of testosterone treatment with or without chest reconstruction surgery (CRS) on mental health in transgender women.	Cross-sectional	Beck Anxiety Index (BAI)<br>Beck Depression Inventory (BDI)<br>Snell Clinical Anger Scale (CAS)<br>Mood and sexuality (open-ended measures)	208	Transgender women	Participants on TRT reported reductions in anxiety, anger, and depression when compared to the untreated group. Moreover, there were open-ended reports of increased happiness in the TRT group.	USA
Gomez-Gil et al (2012)	Sought to evaluate the presence of social distress, anxiety and depression in transgender people. There was an emphasis on hormone usage vs non-hormone usage in the sample.	Cross-sectional	Social anxiety and distress scale (SADs)<br>Hospital anxiety and depression scale (HADs)	187	120 transgender people who had undergone hormone therapy.<br>67 transgender people who had not undergone hormone therapy.<br><br>(113 transgender women, 74 transgender men)	Depression, anxiety and distress scores were significantly higher in those who had NOT undergone hormone therapy indicating that transgender people undergoing hormone therapy had better psychosocial outcomes.	Spain
Gomez-Gil et al (2014)	Evaluated the perceived quality of life (QoL) and GAHT use in transgender people attending a gender identity unit before sex reassignment surgery.	Cross-sectional	WHOQOL-BREF QoL<br>Family APGAR Questionnaire (familial relationship satisfaction)	193	Transgender people on GAHT, not on GAHT, and awaiting genital reassignment surgery	Quality of life and familial support was better for those who were on GAHT. However, those that received surgery had the highest QoL.	Spain
Gooren et al (2013)	To assess functional health and mental wellbeing of kathoeys on GAHT. As well	Cross-sectional	Social Functioning Questionnaire (SFQ)Short Form	60	Kathoeys (44 using hormones)	Hormone use was largely unrelated to functional health or	Thaila nd

	as make an inventory of the non-supervised use of cross-sex hormones among kathoeys.		Health Survey 36 (SF-36)			mental well-being but there was a trend towards somewhat better mental health and vitality in participants not using cross-sex hormones	
Gooren et al (2015)	Compared 60 transgender men (toms) with 60 transgender women (kathoeys) regarding their use of cross-sex hormones, mental wellbeing and acceptance by their family	Cross-sectional	Life Orientation Test Revised (LOT-R) The Social Functioning Questionnaire (SFQ) The Short Form Health Survey 36 (SF-36)	120	60 transgender men 60 transgender women	There were no differences in mental well-being among transgender men and transgender women However, transgender men currently using cross-sex hormones scored on average poorer on mental health measures when compared to non-users. Those undergoing cross-sex hormone treatment were 5-8 times more likely to be associated with poor scores on interpersonal relationships with their parents. Self-acceptance was not significantly different between transgender men and women using cross-sex hormone therapy vs those who were not but they were approaching statistical significance.	Thailand

Gorin-Lazard et al (2012)	Assessed the relationship between hormone therapy and self-reported quality of life scores in transgender people while exploring the confounding factors that may influence this relationship.	Cross-sectional	Beck Depression Inventory (BDI)<br>Short form 36 (SF-36)	61	44 Transgender people who had undergone hormone therapy<br>16 Transgender people who had not undergone hormone therapy.<br><br>(31 transgender women, 30 transgender men)	Hormone therapy has a positive effect on the overall mental and physical health of transgender people. Depression in an independent context was found to be predictive of poorer quality of life regardless of hormone therapy.	France
Gorin-Lazard et al (2013)	Assessed the relationship between hormonal therapy, self-esteem, depression, quality of life (QoL), and global functioning.	Cross-sectional	Self-esteem (Social Self-Esteem Inventory)<br>Mood (Beck Depression Inventory)<br>QoL (Subjective Quality of Life Analysis)<br>Global functioning (Global Assessment of Functioning)	67	MtF (36)<br>FtM (31)	Transgender people undergoing hormonal therapy in the period preceding surgery reported better self-esteem, lower prevalence of depression, and greater QoL mental scores.	France
Grannis et al (2021)	Assessed the effect of gender affirming T treatment on internalizing symptoms, body image dissatisfaction, and activation patterns within the amygdala-prefrontal cortex circuit in transgender adolescent boys.	Cross-sectional	General anxiety (SCARED)<br>Social anxiety (LSAS)<br>Depression (CDI)<br>Suicidality (NSSI)<br>Body image dissatisfaction (BIS)	42	Transgender boys undergoing GAHT (19)<br>Transgender boys not undergoing GAHT (23)	Anxiety, depression and suicidality were lower for the individuals undergoing GAHT when compared to those not using GAHT.	USA

Green et al (2022)	Examined mental health among transgender and non-binary youth who receive GAHT.	Cross-sectional	Depression (PHQ-2)<br>Suicidality (youth risk behavior survey)<br>Social support	11,914	Transgender and non-binary youth on GAHT (9019)<br>Transgender and non-binary youth who did not report being on GAHT (2895)	Use of GAHT was associated with lower odds of recent depression and seriously considering suicide compared to those who wanted GAHT but as of yet had not received the hormone treatments.	USA
Hughto et al (2020)	Examined changes in self-reported suicidal ideation, suicide attempts, and non-suicidal self-injury (NSSI) before and after initiating the gender affirmation process, and linear regression analyses to examine associations between gender affirmation experiences and self-reported depressive, anxiety, and stress symptoms.	Cross-sectional	Transgender related discrimination (adapted from Rood et al 2015)<br>Gender affirmation<br>Investigator created NSSI scale<br>Depression, anxiety, and stress scale (DASS-21)	288	Transgender adults<br><br>234 transmasculine (49 non-binary people)<br>54 transfeminine (4 non-binary people)	Results showed that overall participants reported lower levels of anxiety, depression and stress post HRT.	USA
Jellestad et al (2018)	Examined the associations between gender affirming interventions, depression and quality of life scores in transgender people.	Cross-sectional	Short Form (36) Health Survey questionnaire (SF-36).<br>Short Form of the Center for Epidemiologic Studies-Depression Scale (ADS-K)	143	Transgender people (77 transfeminine, 41 transmasculine, 25 non-binary people).	Both transfeminine and transmasculine individuals reported a lower QoL compared to the general population. Within the trans group, nonbinary individuals showed the lowest QoL scores and significantly more depressive symptoms. Medical	Switzerland

						hormone interventions were associated with better mental wellbeing but even after successful medical transition, transgender people remain a population at risk for low QoL and mental health, and the nonbinary group showed the greatest vulnerability	
Keo-Meier et al (2011)	Examined the relationship of hormone replacement therapy, specifically testosterone, with various mental health outcomes	Cross-sectional	Depression, Anxiety, and Stress Scale (DASS)<br>Multidimensional Scale of Perceived Social Support (MSPSS)<br>Short Form 36-item Questionnaire version 2 (SF-36v2)	369	Transgender men	Results of the study indicate that female-to-male transsexuals who receive testosterone have lower levels of depression, anxiety, and stress, and higher levels of social support and health related quality of life.	USA
Newfield et al (2006)	Evaluated health related quality of life in transgender men on the full SF-36 measurement domains. Of particular note are the depression, anxiety, and role emotional (social functioning) scales.	Cross-sectional	Short form 36-Question Health survey V2 (SF-36v2)	446	Transgender men	Transgender men who had received hormone therapy reported improvements in their mental health, depression and anxiety on the SF-36 when compared to those who had not received hormone therapy. This was also true for their role emotional score (social functioning)	USA

						which improved following GAHT.	
Simbar et al (2018)	Aimed to assess the body image and quality of life of individuals suffering from gender dysphoria (GD) who were undergoing different types of treatment or no treatment at all.	Cross-sectional	Quality of life (WHOQoL-BREF)<br>Body image (Fishers Body Image questionnaire)	90	Untreated (no hormone therapy or surgery) (n = 30)<br>Hormone therapy (n = 30)<br>Gender-reassignment surgery (n = 30)	Both quality of life and body image were significant higher in the GAHT group when compared to the no treatment group.	Iran
Tomita et al (2019)	Explored relationships between three stages of gender affirming medical interventions (GAMIs) and scores on 6 behavioral health symptom measures.	Cross-sectional	10-item Suicidal ideation scale (SIS-10)<br>10-item Center for Epidemiologic Studies Depression Scale-10th edition (CES-D-10)<br>Social phobia inventory (Mini-SPIN)<br>Generalized anxiety disorder (GAD-7)<br>17-item PTSD Check List–Civilian Version (PCL-C)	868	Transgender people (363 transfeminine, 505 transmasculine)	Better depression, anxiety, PTSD, and mental health scores were associated with those who had undergone GAMIs such as hormone therapy and/or surgery.	USA
Tucker et al (2018)	Investigated whether undergoing hormone or surgical transition intervention(s) relates to the frequency of recent suicidal	Cross-sectional	Suicidal behaviors Questionnaire (SBQ-R)<br>Patient Health	206	Transgender veterans (178 transgender women, 28	Veterans who had undergone hormone therapy and other surgical intervention to affirm their gender	USA

	ideation (SI) and symptoms of depression in transgender veterans.		Questionnaire-9 (PHQ-9)		transgender men)	reported significantly lower levels depression.	
Turban et al (2022)	To examine associations between recalled access to gender-affirming hormones (GAH) during adolescence and mental health outcomes among transgender adults in the United States.	Cross-sectional	Psychological distress (Kessler 6)<br>Self-report binge drinking<br>Self-report drug use	21,598	Never accessed GAHT (8860)<br>Early adolescence GAHT - age 14–15 (119)<br>Late adolescence GAHT - age 16–17 (362)<br>Adulthood - age >18 (12257)	Lower levels of psychological distress, binge drinking, and drug use were observed in the group that had access to GAHT the earliest in adolescence (14-17).	USA
Yang et al (2016)	Assessed the quality of life among Chinese transgender women (those using GAHT and those not using GAHT).	Cross-sectional	36-item Short-Form Health Survey (SF-36)	209	Transgender women	Transgender women who used hormone therapy reported significantly lower levels of the physical component summary and the mental component summary.	China

Achille et al (2020)	Examined the associations of endocrine intervention (puberty suppression and/or cross sex hormone therapy) with depression and quality of life scores over time in transgender youths (6-month intervals between 2013-2018).	Prospective cohort	The Center for Epidemiologic Studies Depression Scale (CESD-R), The Patient Health Questionnaire Modified for Teens (PHQ-9_Modified for Teens), Quality of Life Enjoyment and Satisfaction Questionnaire (QLES-Q-SF)	50	Transgender youths (33 transgender men, 17 transgender women)	Mean depression and suicidal ideation scores decreased over time from baseline (no hormones) to study endpoint (substantial hormone intervention)	USA

Aldridge et al (2020)	investigated the effect of 18-month gender affirming hormone therapy (GAHT) on depression and anxiety symptomatology and the predictors on mental health outcomes in a large population of transgender people	Prospective cohort	Hospital Anxiety and Depression Scale (HADS), the Multidimensional Scale of Perceived Social Support (MSPSS) and the Autism Spectrum Quotient—Short Version (AQ-Short)	178	Transgender people (adults and youths) (95 assigned male at birth, 83 assigned female at birth)	Symptomatology was significantly decreased for depression and non-significantly reduced for anxiety. Scores on the MSPSS predicted reduction in depression, while scores on the AQ-Short predicted reduction in anxiety.	UK
Allen et al (2019)	A longitudinal evaluation of the effectiveness of gender affirming hormones for improving psychological well-being and decreasing suicidality among transgender youth referred to a transgender health specialty clinic at a large Midwest children's hospital.	Prospective cohort	The Ask Suicide-Screening Questions (ASQ) The General Well-Being Scale (GWBS) of the Pediatric Quality of Life Inventory	47	Transgender youths (33 assigned female at birth, 14 assigned male at birth)	After gender-affirming hormones, a significant increase in levels of general well-being and a significant decrease in levels of suicidality were observed.	USA

Arnolduss en et al (2022)	Investigated whether different aspects of self-perception changed after GAHT and if there were differences for adolescent transgender men or women.	Prospective cohort	SPPA	70	Transgender adolescents (49 transgender men and 21 transgender women)	Physical appearance and global self-worth improved significantly over the course of gender-affirming hormone treatment. Scholastic competence, social acceptance, athletic competence, and close friendship remained stable over time.	The Nethe rlands
Colizzi et al (2014)	Evaluated the presence of psychiatric conditions in transgender patients and compared psychiatric conditions related to the hormonal intervention in a one year follow-up assessment.	Prospective cohort	Zung Self-Rating Anxiety Scale (SAS)<br>Zung Self-Rating Depression Scale (SDS)<br>Symptom Checklist 90-R (SCL-90-R)	118	Transgender people<br>78 transgender women<br>29 transgender men	Anxiety, depression and psychological distress reduced significantly following GAHT at the 12 month marker for the transgender patients.	Italy
Defreyne et al (2019)	Assessed whether anger intensity increases in transgender men undergoing hormone therapy to identify the predictors for anger intensity. Also transgender women were assessed to see if anger intensity decreases following hormone therapy.	Prospective cohort	Hormone usage over 3 years (baseline, 3 months, 12 months, and 36 months follow ups).<br>STAXI-2 (State-Trait Anger Expression Inventory-2)<br>State Anger (S-Anger)	898	440 Transgender men<br>468 Transgender women	State anger levels are more dependent on psychological vulnerability as opposed to hormone usage.	Belgiu m

Fisher et al (2016)	Assessed whether cross sex hormone therapy related body changes affected psychobiological well-being in people with gender dysphoria.	Prospective cohort	Body Uneasiness Test (BUT)<br>Symptom Checklist 90 revised (SCL-90-R)<br>Gender Identity/Gender Dysphoria questionnaire (GIDYQ-AA)<br>Beck Depression Inventory (BDI) II	413	125 transgender women<br>42 transgender men	Beck depression inventory scores significantly reduced over the course of gender affirming hormone therapy over the two years.	Italy
Heylens et al (2014)	Investigated how gender reassignment therapy affects psychopathology and other psychosocial factors.	Prospective cohort	Symptom Checklist-90 (SCL-90)	57	Individuals with gender dysphoria<br><br>(46 transgender women, 11 transgender men)	Significant decreases were found in the subscales such as anxiety, depression, interpersonal sensitivity, and hostility.	Belgium
Manieri et al (2014)	Described the care and treatment of subjects, highlighting both the endocrine metabolic effects of the hormonal therapy and the quality of life (including interpersonal relationships) during the first year of cross-sex therapy.	Prospective cohort	WHO QoL questionnaire (WHOQOL-100)	83	56 Transgender women<br>27 Transgender men	Most transgender women reported higher emotional lability (exaggerated changes in mood) and sensitivity in interpersonal relationships. Also reported a higher emotional intensity in alignment with life events. Transgender men reported an	Italy

						increase in impulsiveness and aggressiveness.	
Matthys et al (2021)	Assessed the impact of gender affirming hormone treatment on affect among transgender people.	Prospective cohort	Positive and negative affect schedule (PANAS)	873	451 transwomen<br>422 transmen	Negative affect scores significantly decreased after one year and remained consistent across the remaining 24 months.	The Nethe rlands
Mazzoli et al (2022)	Evaluated differences in Autism Spectrum Quotient (AQ) scores between a sample of hormone-naïve transgender and cisgender people and the impact of gender-affirming hormonal treatment (GAHT) on AQ scores across time. Furthermore, assessed alexithymia and social anxiety as possible mediators of changes in AQ scores.	Prospective cohort	Autism spectrum Quotient (AQ)<br>Alexithymia<br>Social anxiety	62	Transgender men (38)<br>Transgender women (24)	Transgender people showed a significant reduction in autism quotient scores over GAHT. Additionally, alexithymia and social anxiety reduced after GAHT.	Italy

Metzger & Boettger (2019)	Aimed to analyze the effect of testosterone therapy on transgender men's personalities.	Prospective cohort	NEO-Personality inventory (NEO-PI-R)	50	23 Transgender men<br>27 Cisgender matched controls	The first three months yielded the biggest changes in personality with neuroticism decreasing along with depression. Conversely, extraversion, assertiveness, and warmth all significantly increased. Interpersonal stress decreased, social interaction traits all increased, and personal wellbeing also increased.	Germany/Switzerland
Motta et al (2018)	Assessed the effect of testosterone treatment on anger expression in transgender men.	Prospective cohort	Spielberger's State-Trait Anger Expression Inventory (STAXI-2)	52	Transgender men	Anger expression was found to be significantly higher following testosterone usage.	Italy

Nobili et al (2020)	Explored the impact of Cross-sex Hormone Treatment (CHT) on autistic traits, independent of changes in anxiety longitudinally.	Prospective cohort	Hospital Anxiety and Depression scale (HADs)<br>AQ Short (Autistic trait scale)	118	Transgender people<br><br>(59 assigned male at birth, 59 assigned female at birth)	There were no significant changes in anxiety scores over the four year period.	UK
Skewis et al (2021)	Aimed to examine the effect of newly commencing GAHT on gender dysphoria and quality of life (QoL) over a 6 month period in comparison to cisgender people (not on GAHT).	Prospective cohort	Gender preoccupation and stability questionnaire (GPSQ)<br>RAND short form 36 (SF-36)	180	77 trans (35 initiating feminizing GAHT, 42 masculinizing GAHT)<br>103 cisgender (53 men, 50 women)	Gender dysphoria decreased following GAHT as well as an observed increase in QoL for transgender participants. Gender dysphoria scores were still higher than cisgender matched controls though.	Australia
Slabbekoorn et al (2001)	Investigated whether cross-sex hormone treatment in transsexuals affected the intensity of negative and positive emotions in general, and aggressive and sexual feelings in particular.	Prospective cohort	Expectancy list of mood and sexual interest<br>The Dutch Sex Role Questionnaire (NSV)<br>The Affect Intensity Measure (AIM)<br>The Short Anger Situation Questionnaire (ASQ)<br>The Affective Communication Test (ACT)	101	47 Transgender women<br>54 Transgender men	Depression, tiredness, tenseness and changeable mood all improved following hormone therapy. However, aspects of negative emotions related to powerlessness, disappointment, and sadness were equal following transgender women's hormone usage. Positive	The Netherlands

						emotions improved for transgender women on measures of happiness and liveliness. Transgender men reported higher levels of anger following GAHT.	
Turan et al (2018)	Examined the alteration of body uneasiness, eating attitudes/behaviors, and psychological symptoms in transgender men after Cross sex Hormone Therapy.	Prospective cohort	Body Uneasiness Test (BUT)<br>Eating Attitudes Test (EAT-40)<br>Symptom Checklist-90-Revised (SCL-90-R)	77	Transgender men<br><br>40 cisgender women (as controls)	SCL-90-R scores decreased at 6 months following cross sex hormone therapy i.e., Interpersonal Sensitivity, Depression, Anxiety, Hostility, Phobic Anxiety etc.	Turke y
Van Goozen et al (1995)	Sought to understand the activating effects of sex hormones on behavioral measures in transgender people.	Prospective cohort	Instruments<br>Aggression and anger proneness questionnaires.<br>Buss-Durkee Hostility inventory<br>The Anger Expression (AX) scale	50	35 Transgender men<br>15 Transgender women	The administration of androgens to females was associated with an increase in aggression proneness, sexual arousability and spatial ability performance.<br>The effects of cross-sex hormones were just as pronounced in the male-to-female group upon androgen deprivation: anger<br>and aggression	The Nethe rlands

						proneness, sexual arousability and spatial ability decreased.	
van Kemenade et al (1989)	Examined the role of GAHT on sexuality, aggression and mood in a sample of transgender women	Prospective cohort	Sexuality Aggression Mood	14	Transgender women (MtF)	Sex function was reduced during the first 4 weeks. Participants felt more relaxed around week 4 of treatment. Feelings of irritation were stable but showed a dip around the 4th week. Anxiety and felt-tension reduced over the four week period too. Items like cheerful, sociable and friendly, gloomy and unhappy, aggressive and changeable were stable during the 8-week period.	The Netherlands

Araya et al (2021)	Aimed to describe adolescents' history and experiences with romantic partners and found aspects of hormone usage that influenced these factors.	Qualitative	Semi-structured interviews	30	Transgender adolescents (18 transgender men and 12 transgender women)	Participants reported increases in their confidence and assertiveness following hormone therapy. Transmasculine individuals also reported undesired outcomes such as a feeling of difficulty controlling anger and feeling emotionally distant.	USA
Giovanardi et al (2019)	Explored the psychological aspects of hormonal treatments for gender non-conforming adults, including the controversial use of puberty suppression treatments.	Qualitative	Semi-structured interviews	10	Adult transgender women	Overall, the participants were satisfied with treatment as it prevented the severe body dysphoria and social phobia trans-people experience with puberty. It seems that the risk of social isolation and psychological suffering is increased by the general lack of acceptance and stigma toward trans-identities in the Italian society. However, during gender transitions, they highlight the need to focus more on internal and psychological aspects, rather than	Italy

						over-emphasize physical appearance.	
Mohamed and Hunter (2019)	Investigated transgender women's experiences and attitudes to hormone therapy.	Qualitative	Beliefs about medicines questionnaire (BMQ) Inductive thematic analysis	67	Transgender women	Improvements in depression were highlighted by participants as well as overall wellbeing and feelings of gender dysphoria.	UK

Riggs et al (2020)	Explored the views of transgender youth and their parents on medical affirming interventions.	Qualitative	Semi-structured interviews	21	10 Transgender youths (5 male, 4 female, 1 non-binary)<br>11 Parents (9 female, 1 male, 1 non-binary)	Hormone blockers were seen to improve aspects of happiness but there was an important distinction made between their mental health affirming effects and the fact that they "just stop puberty"	Australia
Rosenberg et al (2019)	This study aimed to provide an exploration of trans women's negotiation of the psycho and physio-sexual shifts which result from GAHT.	Qualitative	Semi-structured interviews	12	Transgender women	Participants reported a greater sense of liberation following GAHT. One participant talked about how their depression and anxiety had decreased.	Australia
Ussher et al (2022)	Discusses the experience and construction of gender transitioning and gender affirmation for trans women of color living in Australia, associated with the risk of social exclusion or violence.	Qualitative	In depth Semi-structured interviews and photovoice methodology	31	Transgender women of color	Most participants reported that they experienced increased positivity, confidence, and positive mental wellbeing. However, participants reported that hormones were a "daily reminder of being trans" and were associated with some negative emotions such as crying a lot or emotional responses.	Australia
